# Comparison of the problem based learning-driven with the traditional didactic-lecture-based curricula

**DOI:** 10.5116/ijme.5749.80f5

**Published:** 2016-06-12

**Authors:** Muhammad A. Zahid, Ramani Varghese, Ahmed M. Mohammed, Adel K. Ayed

**Affiliations:** 1 Department of Psychiatry, Health Sciences Center, Kuwait University, Kuwait; 2 Assessment Office, Health Sciences Center, Kuwait University, Kuwait; 3 Department of Surgery, Health Sciences Center, Kuwait University, Kuwait

**Keywords:** Problem-based learning, curriculum reforms, psychiatry, Kuwait, K2 type MCQs, curriculum development, medical students, evaluation

## Abstract

**Objectives:**

To compare the Problem-based learning (PBL) with the traditional lecture-based curricula.

**Methods:**

The single best answer Multiple Choice Questions (MCQ) and the Objective Structured Clinical Examination (OSCE) were used to compare performance of the lecture-based curriculum with the PBL medical student groups. The reliability for the MCQs and OSCE was calculated with Kuder-Richardson formula and Cronbach’s alpha, respectively. The content validity of the MCQs and OSCE were tested by the Independent Subject Experts (ISE). The Student’s t-test for independent samples was used to compare the item difficulty of the MCQs and OSCE’s, and the Chi-square test was used to compare the grades between the two student groups.

**Results:**

The PBL students outperformed the old curriculum students in overall grades, theoretical knowledge base (tested with K2 type MCQs) and OSCE. The number of the PBL students with scores between 80-90% (grade B) was significantly (p=0.035) higher while their number with scores between 60 to 69% (grade C) was significantly p=0.001) lower than the old curriculum students. Similarly, the mean MCQ and the OSCE scores of the new curriculum students were significantly higher (p = 0.001 and p = 0.025, respectively) than the old curriculum students. Lastly, the old curriculum students found the K2-MCQs to be more (p = 0.001) difficult than the single correct answer (K1 type) MCQs while no such difference was found by the new curriculum students.

**Conclusions:**

Suitably designed MCQs can be used to tap the higher cognitive knowledge base acquired in the PBL setting.

## Introduction

The Problem-based learning (PBL) model is now a well-established learning method, where students take center stage in case-based, self-directed learning.^1^^,^^2^ It is claimed that the PBL adopts problem-solving approach and goes beyond rote memorization and simple acquisition of knowledge attributed to the traditional didactic lecture-based teaching. In the PBL, the students draw upon their existing knowledge and engage in active learning with particular relevance to the given learning topics as opposed to the passive learning, based on teacher-designed lectures and instructions. Since its introduction more than four decades ago, and subsequent endorsement by the Association of Medical Colleges and the World Federation of Medical Education, the PBL has swept the world of medical education, and the literature is replete with descriptions, developments, and implementation of the PBL-driven curricula.^3^^,^^4^ The studies evaluating the efficacy of the PBL, however, have produced conflicting results.^5^^,^^6^ The PBL-based curriculum has been shown to be more effective in promoting clinical competency but its efficacy in promoting theoretical knowledge base has been questioned, and some studies have even reported deficiencies in the knowledge base in the PBL student subgroups.^1^^,^^6^^,^^7^ Despite its widespread worldwide introduction, the advantages of the PBL over the traditional curriculum have not been categorically established, and both the proponents and opponents of the PBL continue to dispute its merits and advantages over the traditional didactic lecture-based curriculum.^8^  

Numerous systematic reviews and meta-analyses comparing PBL with the traditional curriculum have failed to provide unequivocal support in favor of the PBL.^2^^,^^9^^,^^10^^,^^11^^,^^12^ Although some studies have found PBL to be more effective in promoting clinical skills and professional competency, its usefulness in promoting the broad knowledge remains uncertain.^7^^,^^11^^,^^13^^,^^14^^,^^15^ There are two main reasons for these conflicting findings. First, the PBL is not a uniform curriculum intervention, and as Maudsley^16^ has pointed out, there are different definitions and ways of delivering the PBL, which makes it difficult to isolate the different processes involved in the PBL for comparison purposes. Second, different outcome measures have been used to evaluate its efficacy which makes it difficult to draw firm conclusions, and make comparisons, with the traditional curricula.^11^ Although the multiple choice questions (MCQs) have been widely used to compare traditional with the PBL curriculum, their usefulness as a reliable and valid tool to draw comparisons between the two types of curricula has been questioned. It has been argued that, since the MCQs assess basic cognitive skills including factual knowledge recall and comprehension, they do not measure the deeper cognitive level of the knowledge base attributed to the PBL.^17^^,^^18^ However, it has been claimed that properly designed MCQs can be used to tap the higher cognitive skills including analysis, integration, and synthesis and application, which characterize the type of learning acquired in the PBL setting.^19^^,^^20^^,^^21^^,^^22^  Despite the fact that a number of studies have used MCQs to demonstrate superior performance of the PBL student groups, the usefulness of the MCQs as a reliable and valid tool to measure the higher cognitive level of the broad knowledge-base,  remains uncertain.^1^^,^^6^^,^^11^^,^^23^^,^^24^^,^^25^ Using appropriately designed MCQs, the purpose of this study was to compare the performance of the PBL-driven, with the traditional didactic lecture-based, curricula students.

## Methods

### Setting

The School of Medicine, Kuwait University, admits about 100 students every year and offers a 7-year undergraduate teaching program; 1st year is pre-medical; 2nd to 4th years are pre-clinical, and the 5th to 7th years are clinical. Some students drop out as years progress while some failed students take re-sit exam with the previous year’s class. The faculty curricula, for both preclinical and clinical programs, were reformed in 2005-6. The traditional teacher-centered, didactic lecture-based classroom teaching was replaced with the student-centered, small group, problem-based learning (PBL). The weekly learning objectives (WLOs) for the 8-week psychiatric rotation were identified, and the didactic lectures (N= 24) were replaced with 14 (2 for each case) PBL group meetings. Based on the WLOs, seven PBL cases, comprising psychoses, affective disorders, anxiety disorders, somatoform disorders, organic mental syndrome (delirium), substance abuse, and child psychiatry (attention deficit disorder), were identified. During the daily Case Conference meetings (N=28), the students, under supervision of their respective tutors, made case presentations and demonstrated the clinical findings with live patient interviews.

### Study design

A cross-sectional descriptive study design was used. The data was accessed from the on-site evaluations of both the old and the new curricula students.

### Participants and sample size

The participants included 91 students (48 males; 43 females) of the 2009/2010- year and 69 students (48 males; 43 females) of the 2011/2012- academic year. The old curriculum students included 16 re-sits while 25 students had dropped out from the new curriculum class. All the old curriculum students were examined only once at the end of the academic year while the new students were examined twice; once at the end of the 8-week psychiatry rotation, and then during the annual examination.

The institutional scientific and research committee was approached for ethical approval which allowed the study to proceed without any further review as it did not involve human subjects or animals and the study data consisted of students’ performance in examinations.

### Description of the intervention

In accordance with the weekly learning objectives, a total number of seven PBL cases were identified. Each PBL case was discussed in two sessions. A typical PBL group, comprising 5 to 6 students, began the session (one) with analysis of the unfamiliar terms and defining the stimulus (case trigger) in a concise statement (describing the nature of the disorder) followed by the brainstorming session. Drawing upon their pre-existing knowledge and engaging in the process of elaboration, the students brought forth different hypotheses to explain the possible underlying (psychopathological) mechanisms for the given clinical case manifestations. This exercise helped students identify gaps in their knowledge in ascertaining clinical manifestations, differential diagnoses, and management of the given case. The students then dispersed to engage in self-directed learning. Using a variety of resources, they sought out and learned additional information which helped to bridge the gaps in their pre-existing knowledge. Additional information containing details of history, clinical manifestations, and investigations was sent to them during this time. In addition, the students clerked and presented patients with similar manifestations during the once daily Tutor-supervised Clerkship sessions and the Case Conferences. This helped them to narrow down the hypotheses (generated during the previous group meeting) to the main diagnosis and formulate the individual management plans for the case. The second PBL group meeting, called the Reporting back session, was conducted six days later. It began with 5-7 minute learning topic presentations by the students. Then, the case review was carried out in which the underlying mechanisms (psychopathology) for the given clinical manifestations were discussed and the management plan formulated. The session ended with the process review when the group members reflected on the productivity of the PBL sessions. The role of the PBL facilitator, a subject specialist, was limited to maintenance of cooperative, productive, and positive group environment. Using his content knowledge sparingly, he helped foster receptive and non-judgmental group culture, encouraged free and frank expression by all group students and ensured that the group remained focused.

**Table 1:** Comparison of grades between new and old curriculum students

Grades^*^		2012 (new) N=69		2010 (old) N=91		^**^p-value
		Number	%	Number	%	
A	90%-100%	3	4.35	3	3.30	0.941
B	80-89%	23	33.3	16	18.4	0.035
C+	70-79%	38	55	42	46	0.338
C	60-69%	5	7.2	26	29	0.001

*Four students failed in old curriculum; **Chi-square test

### Assessment methods

The 2009-10 assessment included 220 MCQs and eight OSCE stations. The 2011-12 examination consisted of 225 MCQs and ten OSCE stations.

### MCQs

It has been argued that MCQs most often test basic cognitive level of the knowledge-base, namely the factual information recall and comprehension with little consideration for the degree or depth of cognitive level of the learning involved.^17^ Based on their discriminating power to tap the higher cognitive level and deeper understanding of the knowledge-base, the MCQs have been classified into K1/K2 type. The K1 questions require candidates to recognize, remember, retrieve, identify, and recall a term while in the K2 type questions, the candidates have to select the reasons or identify the most correct explanation for the given clinical manifestations described in the question.^18^ The K1 type questions require selection of the single correct answer out of the given five options while the K2 type questions require identification of the most correct answer out of the five (all correct) options depicting a continuum containing correct answers albeit with varying degrees of correctness (see Appendix). . It has been claimed that the K 2 type MCQs test the depth, integration, analysis, and application of knowledge in varied clinical situations while the KI type questions test simple recall of facts or basic comprehension.^18^^,^^25^^,^^26^^,^^27^^,^^28^

### OSCE

The clinical skills and professional competency were assessed with the 7-minute couplet-OSCE stations. The clinical competency level of the OSCE stations conformed to the course objectives and covered a variety of clinical problems including insomnia, suicidal risk, cognitive impairment, delirium, depressed mood, hallucinating patient, panic disorder, somatization disorder, psychoeducation and counselling for patients and their families. Each station was followed by a couplet, pen-and-pencil station in which students were required to answer questions concerning diagnosis/management of the clinical problem, assessed in the previous station.

**Table 2:** Comparison of mean scores between new and old curriculum students

Items	Mean ± SD		^*^p-value
	2012 (new)	2010 (old)	
MCQ	67.92±9.7	61.11±6.8	0.001
OSCE	79.91±5.1	76.91±10.1	0.025

*Student’s t-test for independent samples

### Procedures

#### K1/K2 MCQ categorization

Independent subject experts (renowned senior professors visiting as external examiners) cross-checked the MCQs and categorized them into K1 or K2 type. Most (104/225, 79.6%) of the new curriculum questions were classified as K2 type while most (144/220, 80.0%) of the old curriculum questions were categorized as K1 type. The performance of the students on both K1 and K2 type MCQs was compared to see if performance differed in the two groups.

#### Reliability of assessment procedures

The MCQs internal reliability coefficient, indicator of the homogeneity of test items,^29^ was calculated with Kuder-Richardson formula (KR-20) while the OSCE’s internal reliability was calculated with the Cronbach’s alpha. The validity of the assessment procedures was established by the Independent Subject Experts’ (ISE) pre-assessment review of the subject matter of both MCQs and the OSCE stations. The post-test item analysis was performed to look for indications of miss-keyed items or items with writing flaws.

#### Statistical analysis

The data were analyzed on SPSS, version 20.  The Student’s t-test for independent samples was used to compare the post-hoc mean item difficulties between the old and new curricula MCQ scores The Chi-square tests were used to compare the grades for the two groups of students.

**Table 3:** Comparison of K1/K2 type questions’ distribution and the measured item difficulty between new and the old curricula students’ performance in the MCQs

Curriculum	n^*^	Measured Item Difficulty				^**^p-value
		K1-Items		K2-Items		
		n^*^	Mean ± SD	n^*^	Mean ± SD	
Old	220	144	0.68 ± 0.24	76	0.56 ± 0.26	0.001
New	225	121	0.67 ± 0.26	104	0.64 ± 0.25	0.545
p-value			0.693		0.036	

*Number of MCQs; **Student’s t-test for independent samples

## Results

### Reliability of assessment procedures

The reliability coefficients of the new and the old curricula MCQs were 0.8 and 0.72 respectively, which lie well within the acceptable range. The Cronbach’s alpha values for the OSCE were 0.75 and 0.82, for the new and the old curriculum, respectively, which again were well within acceptable limits. 

### Comparison of grades

The number of the PBL students with scores between 80-90% (grade B) was significantly (p = 0.035) higher while their number with scores between 60 to 69% (grade C) was significantly (p = 0.001) lower than the old curriculum students (Table 1). Similarly the mean MCQ and the OSCE scores of the new curriculum students were significantly higher (p = 0.001 and p = 0.05, respectively) than the old curriculum students (Table 2). The female students’ scores were higher than the males in both new and the old curricula students, but the difference was not statistically significant.

### Comparison of MCQ scores

The post-test item analysis showed that the old curriculum students found K2-questions to be significantly (p = 0.001) more difficult than K1-questions while no such difference was found by the new curriculum students (Table 3). In other words, 56% of the old and 64% of the new curricula students answered the K2 type questions correctly (p = 0.036) while 68% of the old and 67% of the new curricula students answered the KI type questions correctly.

## Discussion

Our findings suggest that the PBL-based curriculum students performed significantly better than the didactic lecture-based curriculum students both in theoretical knowledge base (K2 type MCQs) and clinical examination (OSCE). The proportion of the new curriculum students with top grades was significantly higher while their number with lower grades was significantly less than the lecture-based curriculum students. Moreover, the old curriculum students found K2 questions to be more difficult while the new curriculum students found no such difference between the K2 and K1 type questions. Similarly, the mean OSCE scores of the new curriculum students were significantly higher than the old curriculum students.

The PBL-based curriculum has been shown to be more effective in promoting clinical competency with little or no impact on the theoretical knowledge base.^10^^,^^23^ In fact, the PBL student groups have been reported to do less well on the overall theoretical knowledge outcomes than the traditional curriculum student groups.^24^ Our new curriculum students outperformed the old curriculum students in both the K 2 type MCQs and the OSCE scores (p = 0.036 and p = 0.05 respectively) suggesting that the PBL process had led to improvement in both dimensions, namely the higher cognitive knowledge base and clinical competency. Our findings are supported by a number of previous reports suggesting that knowledge and clinical problem-solving skills are inextricably related, and that superior performance in one complements the scores in the other.^30^ The assessments of clinical competence have demonstrated that performance is context, or case specific and that the context or case specific knowledge, acquired in the PBL setting improves the ability to identify and manage clinical problems.^31^ Similarly, the scores on multiple-choice examinations have been shown to have positive correlations with performance on assessments of clinical competence,^9^ suggesting that knowledge and clinical competency are closely related. The superior performance of our PBL students in both the theoretical knowledge base and the clinical competency can, therefore, be attributed to the higher cognitive level and deeper understanding of the subject matter, acquired during the PBL processes. 

Since the K2 type MCQs measure comprehension, analysis, integration, and application of knowledge,^19^ the higher K2 type MCQ scores of our PBL students reflect higher cognitive level of their knowledge base. In the PBL model, learners are presented with a (trigger) problem for the initial group discussion. Drawing upon their pre-existing knowledge, the learners elaborate in small groups and construct multiple hypotheses aimed at explaining the underlying mechanisms for the given problem.  According to the ‘situational interest’ hypothesis, this ‘brainstorming’ session involving activation of prior knowledge and elaboration, helps students to identify gaps in their knowledge, a phenomenon termed as ‘Cognitively induced experience of knowledge deprivation’.^32^^,^^33^ The resultant state of knowledge deprivation initiates information seeking behavior in the learners which helps to bridge the gap in their knowledge.^34^ During the students’ pursuit for self-directed, independent  learning,  new information is blended into their prior knowledge, which  is further refined and perfected.^15^ Employing a variety of cognitive processes including elaboration, analysis, integration, application, and critical appraisal, the students engage in the process of ‘narrowing down’ of the hypotheses, generated in the previous group meeting. The process of consolidating some while discarding others forms the very basis of higher cognitive learning attributed to the PBL setting.^15^ The knowledge thus acquired can only be tapped with the single best answer, K2 type, MCQs, with require selection of the most correct while disregarding the least or lesser correct options.^25^^,^^26^^,^^27^

Our findings are consistent with the previous studies reporting significant improvement in the PBL-driven  curriculum students’ deeper understanding of the knowledge base; greater student satisfaction, and more positive student attitudes and perceptions of education.^1^^,^^6^^,^^11^^,^^20^^,^^23^^,^^24^^,^^25^^,^^35^^,^^36^ However, our findings differ from those of the previous studies reporting no difference or even negative effect of PBL on the students’ broad knowledge base.^2^^,^^6^^,^^35^ We believe that the one-time, short-term knowledge retention-focused tests, used in at least some of these studies, are less well suited to measure the deeper understanding and higher cognitive level of the knowledge-base, attributed to the PBL-driven curricula. The traditional curriculum students are adept at preparing thoroughly enough to outscore the PBL students in the tests measuring the simple factual recall of the theoretical knowledge. ^37^

### Limitations of the study

It is important to mention some shortcomings of the study here. First, although mainly driven by PBL, the delivery of our curriculum involved complementary teaching activities including the tutor-supervised, small group clerkship sessions; teaching OSCE sessions; and the Case Conferences, which served as additional learning forums for the students. The PBL has generally been construed as a general construct with little consideration for the complexity of its implementation and the multiple factors likely to affect the outcome of this approach.^38^^,^^39^^,^^40^ The PBL cases, the tutor-supervised clerkship sessions, and the Case Conferences were all synchronized to cover the given Leaning Objectives for the week. The higher MCQ and OSCE scores of our new curriculum students may not necessarily reflect the knowledge and clinical skills acquired during the PBL group meetings alone. The positive effect size of our findings may, therefore, be spurious due to the collateral input from the concomitant teaching methods. However, we believe that the process of synchronization involving multidimensional learning approach promotes the process of elaboration, ^32^^,^^33^^,^^34^  the hallmark of the students’ learning in the PBL setting. Second, the categorization of the MCQs into K1/K2 type may have been relatively arbitrary due to the academic staff bias and ascertaining the cognitive level of the students’ broad knowledge base on the basis of the K1/K2 question types, therefore, may have been inaccurate. However, the MCQs were developed in accordance with the blueprint derived from the WLO’s and were blindly cross checked by the independent subject experts. Finally, the superior performance of our new curriculum students in the OSCE may simply be due to the fact that, following its introduction two years earlier, both the faculty and students, had become ‘familiar’ with the assessment tool.  On the other hand, it can be argued that the superior performance of our students in the OSCE was consistent with the previous reports suggesting that superior performance in clinical situations is closely linked to the ‘case or context specific knowledge’ acquired in the PBL settings.^9^^,^^10^^,^^11^

In summary our experience of replacing the traditional didactic lecture-based with the PBL-driven, curriculum has shown promising results. The synchronization of the different teaching methods, namely aligning the tutor-supervised clerkship sessions and the Case Conferences with the PBL case for the given week, in addition to promoting the PBL processes, resulted in deeper understanding, and superior performance, of our students in the broad knowledge base and clinical competency. This study contributes to the understanding of different educational approaches and describes the usefulness of K2 type MCQs as a reliable and valid tool to evaluate efficacy of the PBL. In particular, it suggests a novel approach to compare the outcome of the PBL with the traditional lecture-based teaching in undergraduate medical students. Further methodologically sound studies are needed to establish the usefulness of the K2 type MCQs to measure the higher cognitive level and deeper understanding of the broad knowledge base, attributed to the PBL curricula.

### Conflict of Interest

The authors declare that they have no conflict of interest.

## Supplementary material

Supplementary file 1 K1/K2 type multiple choice questions
