# Supplementary material for: Comparison of the problem based learning-driven with the traditional didactic-lecture-based curricula
Source: Int J Med Educ. 2016 Jun 12;7:181–7. doi: 10.5116/ijme.5749.80f5 (PMC4912697; doi:10.5116/ijme.5749.80f5)
Supplement: Supplementary file 1 — K1/K2 type multiple choice questions [file ijme-7-181-S1.pdf]

Appendix

K1/K2 type multiple choice questions

Sample old K1 type MCQs

Third person auditory hallucinations

A. are the commonest form of hallucinations in psychoses
B. suggest diagnosis of schizophrenia
C. do not occur in mania
D. are common in organic mental syndromes
E. indicate poor prognosis in schizophrenia

Obsessional ruminations are:

A. always repetitive
B. always unpleasant
C. always unwelcome
D. always accompanied by anxiety
E. all the above

Down's syndrome is significantly associated with development of

A. psychopathic personality
B. schizophrenia
C. social phobia
D. Alzheimer's disease
E. enuresis

Sample new K2 type MCQs

A second-year university student, having failed during the two consecutive years, has been expelled from the college. He attributes his failure to the incompetency of his teachers saying that he was powerless to stop it. What is the most likely explanation of his symptom?

A. Reduced attention span
B. Short term memory impairment
C. First rank symptom of schizophrenia
D. Lack of drive and determination
E. Second rank symptoms of schizophrenia

A 34-year civil servant has brought his wife for constantly bothering him to make repeated checks to ensure that the house entrance doors and windows have been properly locked. He describes the marriage as happy and admires her for conscientiousness and also for keeping the house exceptionally clean. What best explains her problem?

A. Agoraphobia
B. Excessive worrying (Anxiety disorder)
C. Obsessive-compulsive disorder
D. Delusions about her husband’s unfaithfulness
E. Persecutory ideas

A 37-year old gentleman with trisomy 21 syndrome has been increasingly forgetful. He makes frequent mistakes when counting change at the grocery store where he has worked for several years. In the past, he used to perform this task without difficulty. He often cannot recall the names of common objects, and he has started annoying customers with his intrusive questions. What is the most likely diagnosis?

A. Pseudodementia
B. Hypothalamic tumor
C. Alzheimer disease
D. Wilson disease
E. Thiamine deficiency
